# Supplementary material for: Collective dynamics of capacity-constrained ride-pooling fleets
Source: Sci Rep. 2022 Jun 27;12:10880. doi: 10.1038/s41598-022-14960-x (PMC9237121; doi:10.1038/s41598-022-14960-x)
Supplement: Supplementary file 1 — Supplementary Information. [file 41598_2022_14960_MOESM1_ESM.pdf]

Supplementary Material
accompanying the manuscript
Collective dynamics of capacity-constrained ride-pooling fleets

Robin M. Zech,[1] Nora Molkenthin,[2] Marc Timme,[1] and Malte Schröder[1]

[1] *Chair for Network Dynamics, Center for Advancing Electronics Dresden (cfaed) and Institute for Theoretical Physics, Technische Universität Dresden, 01062 Dresden, Germany*

[2] *Complexity Science, Potsdam Institute for Climate Impact Research - Member of the Leibniz Association, 14473 Potsdam, Germany*

I. SUPPLEMENTARY NOTE 1: MINIMAL MODEL QUEUEING THEORY

Consider a minimal model of a ride-pooling service on a network with $N = 2$ nodes $i \in \{1, 2\}$ with distance $l_{1,2} = \langle l \rangle$ with a single vehicle $B = 1$ with capacity θ driving back and forth between the nodes with constant velocity v. Requests follow a Poisson process with rate λ independently and uniformly randomly from one node of the network to the other, i.e. requests appear with rate $\lambda/2$ independently at each node. The travel time for customers is constant since detours are impossible in the minimal topology. Any reduction in efficiency is due to waiting times of customers until they are picked up by the vehicle.

Since all customers currently on the bus are dropped off when the bus arrives at a node, the bus always begins a trip with its full capacity θ available. The dynamics of both nodes are symmetric and it is sufficient to consider the queueing dynamics at a single node.

In the limit of high load ($x \gg 1$ or $x \to \theta$), when the bus is almost never idle since there are always pending requests to be served, the bus departs from node 1 with up to θ of all $q_1(t_k)$ waiting passengers. It returns after a round-trip time at $T_{k+1} = t_k + \Delta t = t_k + 2\,\langle l \rangle / v$. During this time, $z_1(t_k)$ new requests have arrived at node 1 following a Poisson distribution with average $\lambda\,\langle l \rangle / v = x$. The bus again picks up up to θ of the now waiting $q_1(t_{k+1})$ customers and repeats the cycle. The queueing dynamics at the node is described by a queue with Poisson arrivals with rate $\lambda/2$ with deterministic service interval $\Delta t = 2\,\langle l \rangle / v$ and a single server $B = 1$ with batch service with capacity θ [1].

A. Average queue length and ride-pooling efficiency

To compute the ride-pooling efficiency $E = x/\langle C \rangle$, we need to compute the average number of scheduled customers $\langle C \rangle$, consisting of the currently waiting customers at each node, $2\,\langle q \rangle$, and the customers currently on board of the vehicle, x, where $\langle q \rangle$ denotes the time-averaged queue length of the $M/D^\theta/1$-queue at a single node.

Following the calculation of [1], let $\mathbf{p}$ denote the (infinite dimensional) vector of probabilities p_q to observe a queue length q just before the bus arrives. In equilibrium, $\mathbf{p}$ satisfies the fixed point equation

$$\mathbf{p} = P\mathbf{p}\,, \tag{S1}$$

where P is a matrix of transition probabilities with $P_{qq'}$ denoting the probability to observe a queue length q when the queue had length q' at the last service interval. The entries $P_{qq'}$ are Poisson probabilities of the form

$$P_{qq'} = \begin{cases} 0 & \text{if} \quad q < q' - \theta \\ \frac{x^q\, e^{-x}}{q!} & \text{if} \quad q' \le \theta \\ \frac{x^{[q-(q'-\theta)]}\, e^{-x}}{[q-(q'-\theta)]!} & \text{else} \end{cases} \tag{S2}$$

Further calculation [1] yields the probability generating function $G(z) = \langle z^q \rangle$ of $\mathbf{p}$,

$$G(z) = \frac{(\theta - x)(z-1)\prod_{i=k}^{\theta-1}(z - z_k)/(1 - z_k)}{z^\theta e^{x(1-z)} - 1}, \tag{S3}$$

where

$$z_k = -\frac{\theta}{x} \cdot W_0\left(-\frac{x}{\theta} \cdot \exp\left(-\frac{x + 2\pi k i}{\theta}\right)\right) \tag{S4}$$

are the $\theta - 1$ complex zeros of

$$z^\theta e^{x(1-z)} - 1 = 0 \tag{S5}$$

within and on the unit circle and W_0 denotes the principal branch of the Lambert W function. From the probability generating function Eq. (S3), the average queue length $\bar{q}$ *just before the bus arrives at the node* follows as

$$\bar{q} = \frac{\theta - (\theta - x)^2}{2(\theta - x)} + \sum_{k=1}^{\theta-1} \frac{1}{1 - z_k} . \tag{S6}$$

Since on average x customers arrive in one service interval, the average queue length $\underline{q}$ *just after the bus has departed* from the node is $\underline{q} = \bar{q} - x$. From these queue lengths and the Poisson arrival process, it follows that the time-average $\langle q \rangle$ of the queue length is

$$\langle q \rangle = \frac{\bar{q} + \underline{q}}{2} = \bar{q} - \frac{x}{2} = \frac{\theta}{2}\left(\frac{1}{\theta - x} - 1\right) + \sum_{k=1}^{\theta-1} \frac{1}{1 - z_k} . \tag{S7}$$

This expression captures the divergence of the queue length as the system overloads when $x \to \theta$.

With this expression for the average queue length we obtain the average number of scheduled cusomters as $\langle C \rangle = x + 2\langle q \rangle$ and find the expression for the efficiency of the service,

$$E = \frac{x}{\langle C \rangle} = \frac{1}{1 + 2\langle q \rangle / x} , \tag{S8}$$

in the limit of sufficiently large x when the vehicles are not idle.

The above calculations directly transfer to a system with a larger fleet $B > 1$ under the assumption that the vehicles are equidistantly distributed. At constant x, the interval between two vehicles arriving decreases by a factor B and the request rate increases by a factor B, resulting in the same average number x of requests per service interval. Thus, since the number of queued customers does not change, the average number of scheduled customers *per vehicle* becomes $\langle C \rangle = x + 2\langle q \rangle / B$. The efficiency consequently follows [Eq. (6) in the main manuscript]

$$E = \frac{1}{1 + 2\langle q \rangle / (xB)} . \tag{S9}$$

The assumption of equidistant vehicles does not hold exactly in practice. If a single vehicle is delayed, a large number of customers making a request in the interval until the delayed vehicle arrives experience an increase of the waiting time. Fewer customers requesting a ride in the time interval until the next vehicle arrives experience a shorter waiting time. Overall, the average waiting time increases. Consequently, any deviation from an equidistant distribution of vehicles results in lower efficiency than predicted.

This calculation underlies the results presented in Fig. 4a in the main manuscript

B. Estimation of p_{delay}

The queueing theory description also provides a way to compute the probability p_{delay} that a request is delayed due to the capacity constraints. We again consider only a single node due to the symmetry of the dynamics and denote the four relevant discrete random variables, measured when a vehicle arrives at the node, as follows

- Z denotes the number of newly scheduled customers since last service.
- D denotes the number of customers (out of the Z new ones) which cannot be served by the next bus arriving because the capacity constraint would be violated.
- Q denotes the length of the queue just before the bus arrives at the stop.
- Q' denotes the queue length just before the *previous bus* arrived at the stop.

p_{delay} is then defined as the fraction of delayed requests,

$$p_{\text{delay}} = \frac{E(D)}{E(Z)} , \tag{S10}$$

where $E(\cdot)$ denotes the expectation value. The number of newly arriving customers Z is a Poisson random variable with expected value $E(Z) = \lambda \langle l \rangle /(vB) = x$, assuming an equidistant distribution of vehicles as above.

In order to compute the expectation value $E(D)$ of the number of delayed customers, we compute the marginal probability mass function $P(D = d)$ as the sum over the joint distribution for all possible values of Z, Q and Q'

$$\begin{aligned} P(D=d) &= \sum_{z=1}^{\infty}\sum_{q=1}^{\infty}\sum_{q'=0}^{\infty} P(D=d, Q=q, Z=z, Q'=q') \\ &= \sum_{z=1}^{\infty}\sum_{q=1}^{\infty}\sum_{q'=0}^{\infty} P(D=d|Q=q, Z=z, Q'=q')\, P(Q=q|Z=z, Q'=q')\, P(Z=z|Q'=q')\, P(Q'=q')\,. \end{aligned} \tag{S11}$$

The last probability $P(Q' = q')$ is directly given by the equilibrium queue length distribution $p_{q'}$ Eq. (S1)

$$P(Q'=q') = p_{q'}\,. \tag{S12}$$

The number of arriving customers Z is independent of the current state of the queue such that

$$P(Z=z|Q'=q') = P(Z=z) = k_z\,. \tag{S13}$$

If the newly arriving customers Z and the previous queue length Q' are known, Q follows deterministically. The previous vehicle picked up up to θ customers from the Q' waiting customers and Z new customers arrived (compare Eq. (S2). We thus have

$$Q = \begin{cases} Z & \text{if} \quad Q' \le \theta \\ Q' - \theta + Z & \text{else} \end{cases} \tag{S14}$$

customers in the queue and the probability reduces to

$$P(Q=q|Z=z, Q'=q') = \begin{cases} \delta_{q,z} & \text{if} \quad q' \le \theta \\ \delta_{q,(z+(q'-\theta))} & \text{if} \quad q' > \theta \end{cases} \tag{S15}$$

with the Kronecker delta $\delta_{i,j} = 1$ if and only if $i = j$.

The number D of delayed customers follows similarly in three cases:

i) If $Q' \le \theta$, then $Q = Z$. Then $D = \max[0, Z - \theta]$ customers are going to be delayed to a later vehicles.

ii) If $\theta < Q' < 2\theta$, there are $Q' - \theta < \theta$ customers remaining in the queue after the previous vehicle leaves that will be picked up by the next vehicle. From the newly arrived Z requests, $\theta - (Q' - \theta)$ will also be served, whereas $D = \max[0, Z - (\theta - (Q' - \theta))] = \max[0, Z + Q' - 2\theta]$ customers are delayed further.

iii) If $Q' \ge 2\theta$, only requests which where in the queue previously are served by the next vehicle and all the new requests are delayed, $D = Z$.

The relevant conditional probability for delaying customers follows as

$$P(D=d|Q=q, Z=z, Q'=q') = \begin{cases} \delta_{d,\max[0,z-\theta]} & \text{if} \quad q' \le \theta \\ \delta_{d,\max[0,z+q'-2\theta]} & \text{if} \quad \theta < q' < 2\theta \\ \delta_{d,z} & \text{if} \quad q' \ge 2\theta \end{cases} \tag{S16}$$

With Eq. (S11), splitting the summation over q' into the three distinct cases yields

$$\begin{aligned} E(D) &= \sum_{d=0}^{\infty} d\, P(D=d) \\ &= \sum_{d=1}^{\infty}\sum_{z=1}^{\infty}\sum_{q=1}^{\infty} \Bigg[\sum_{q'=0}^{\theta} d\, \delta_{d,\max[0,z-\theta]}\, \delta_{q,z}\, k_z\, p_{q'} \\ &\qquad + \sum_{q'=\theta+1}^{2\theta-1} d\, \delta_{d,\max[0,z+q'-2\theta]}\, \delta_{q,(z+(q'-\theta))}\, k_z\, p_{q'} \\ &\qquad + \sum_{q'=2\theta}^{\infty} d\, \delta_{d,z}\, \delta_{q,(z+(q'-\theta))}\, k_z\, p_{q'} \Bigg]\,. \end{aligned} \tag{S17}$$

Eliminating all terms with $d = 0$ by adjusting the z bounds and evaluating the sum over d yields

$$\begin{aligned} E(D) \; = \; & \sum_{z=\theta+1}^{\infty} \sum_{q=1}^{\infty} \sum_{q'=0}^{\theta} (z-\theta)\, \delta_{q,z}\, k_z\, p_{q'} \\ & + \sum_{q'=\theta+1}^{2\theta-1} \sum_{z=2\theta-q'}^{\infty} \sum_{q=0}^{\infty} (z+q'-2\theta)\, \delta_{q,z}\, k_z\, p_{q'} \\ & + \sum_{z=1}^{\infty} \sum_{q=1}^{\infty} \sum_{q'=2\theta}^{\infty} z\, \delta_{q,(z+(q'-\theta))}\, k_z\, p_{q'} \, . \end{aligned} \tag{S18}$$

For each constellation of (z, q') there is exactly one q within the summation bounds that satisfies $\delta_{q,\cdot} = 1$ in each term such that

$$E(D) = \sum_{z=\theta+1}^{\infty} \sum_{q'=0}^{\theta} (z-\theta)\, k_z\, p_{q'} \; + \sum_{q'=\theta+1}^{2\theta-1} \sum_{z=2\theta-q'}^{\infty} (z+q'-2\theta)\, k_z\, p_{q'} + \sum_{z=1}^{\infty} \sum_{q'=2\theta}^{\infty} z\, k_z\, p_{q'} . \tag{S19}$$

Replacing the infinite sum in the last term using the normalization condition $\sum_{q'=0}^{\infty} p_{q'} = 1$ simplifies the expression

$$p_{\text{delay}} = \sum_{z=\theta+1}^{\infty} (z-\theta)\, k_z \sum_{q'=0}^{\theta} p_{q'} \; + \sum_{q'=\theta+1}^{2\theta-1} \sum_{z=2\theta-q'}^{\infty} (z+q'-2\theta)\, k_z\, p_{q'} + \left(1 - \sum_{q'=0}^{2\theta-1} p_{q'}\right) , \tag{S20}$$

such that only the first probabilities $p_{q'}$ for $q' \leq 2\theta - 1$ are required to evaluate the expression. To evaluate this expression numerically, we cut off the summation over z at $z_{\text{max}} = 50$ (compared to typical values of θ and x less than ten) because of the sharp decay of the Poisson probability k_z.

This calculation underlies the analytical results presented in Fig. 4b in the main manuscript.

II. SUPPLEMENTARY NOTE 2: MEAN FIELD QUEUEING THEORY FOR ARBITRARY NETWORKS

The general idea of the queueing theoretical calculations above can be extended to arbitrary networks with a mean field approach. Assuming the queueing dynamics at all nodes and all vehicles are effectively identical, we can map the above calculation to arbitrary networks with effective parameters. The more heterogeneous the setting in terms of network topology or demand distribution, the larger the deviations from these mean field assumptions.

There are three main differences to the minimal model calculations:

- A single vehicle receives $\lambda/B = xv/\langle l\rangle$ requests per time interval. With the *average distance between nodes* $\langle e\rangle$ (mean edge length), the vehicle receives on average $x\,\langle e\rangle/\langle l\rangle$ new requests between stops. For large fleet sizes and sufficiently low delay-probability p_{delay}, only requests originating at the next stop of the vehicle will be assigned to it. Thus, the expected number $E(Z)$ of newly arrived customers at the next node on its route is

$$E(Z) = \sum_{z=0}^{\infty} k_z = x\,\frac{\langle e\rangle}{\langle l\rangle} =: x_{\mathrm{eff}} \leq x \tag{S21}$$

  where x_{eff} denotes the effective load parameter for the mean field calculations.

- Additionally, in large networks, the inter-arrival times between vehicles at a node are not identical. The inter-arrival time distribution is often more similar to an exponential distribution, reflecting a large number of (almost) independent shortest paths along which vehicles can arrive at a node. We include this variable inter-arrival time by modifying the probability $P(Z=z)$ with an integral over all possible inter-arrival times Δt

$$P(Z=z) = k_z^{\mathrm{exp}} = \int_0^{\infty} e^{-\Delta t}\,\frac{e^{-x_{\mathrm{eff}}\Delta t}\,(x_{\mathrm{eff}}\,\Delta t)^z}{z!}\,\mathrm{d}\Delta t = \frac{x_{\mathrm{eff}}^z\,\Gamma(z+1)}{z!\,(1+x_{\mathrm{eff}})^{z+1}}\,. \tag{S22}$$

  The following calculations are independent of the exact choice of the inter-arrival time distributions as it only enters via $P(Z=z)$.

- Finally, not all passengers are dropped of at every stop such that vehicles do not always have its full capacity θ available for the new requests. We thus have to track the occupancy statistics of the vehicles in addition to the queue length statistics at the node.

Let O denote an additional random variable describing the occupancy of a vehicle at a node immediately after it has dropped of all passengers. The vehicle then has $\theta - O$ seats available for requests from that node. Let $\mathbf{p}$ with entries p_q denote the probability to observe a queue length q (as above) and π with entries π_o denote the probability to observe an occupancy o. Similarly to Eq. (S1) above, we assume a steady state where both probability distributions fulfill the fixed point equations

$$\begin{aligned}\mathbf{p} &= P\,\mathbf{p}\\ \pi &= \Pi\,\pi\,.\end{aligned} \tag{S23}$$

Note that these equations are coupled since the occupancy depends on the number of queued customers and the number of queued (and delayed) customers depends on the occupancy.

To compute the transition probabilities, we neglect correlations between observables that go beyond one service interval. The transition probabilities P for the queue lengths follow similar to the minimal model. We define the relevant random variables as above:

- Z denotes the number of newly arrived customers since last service.
- Q denotes the length of the queue just before the vehicle arrives at the stop.
- Q' denotes the queue length just before the *previous vehicle* arrived at the stop.
- O' denotes the occupancy of the last vehicle that arrived at the node.

The transitions probability is given as the marginal probability

$$P_{q,q'} = P(Q=q\,|\,Q'=q') = \sum_{z=0}^{\infty}\sum_{o'=0}^{\theta} P(Q=q\,|\,Z=z,O'=o',Q'=q')\,P(Z=z\,|\,O'=o',Q'=q')\,P(O'=o'\,|\,Q'=q')\,. \tag{S24}$$

We readily insert

$$P(Z = z \,|\, O' = o', Q' = q') = P(Z = z) = k_z \tag{S25}$$

and

$$P(O' = o' \,|\, Q' = q') = \pi_{o'} \,, \tag{S26}$$

where the latter equation implicitly assumes that there is no correlation between the occupancy of the previous vehicle and the queue length at that time. With given O', Q' and z, the queue length Q is deterministic as

$$Q = \begin{cases} Z & \text{if} \quad Q' \leq \theta - O' \\ Q' - (\theta - O') + Z & \text{else} \end{cases} \,, \tag{S27}$$

resulting in

$$\begin{aligned} P_{q,q'} &= \sum_{z=0}^{\infty} \sum_{o'=0}^{\theta - q'} \delta_{q,z} k_z \, \pi_{o'} + \sum_{z=0}^{\infty} \sum_{o'=\theta-q'+1}^{\theta} \delta_{z,q-q'+(\theta-o')} k_z \, \pi_{o'} \\ &= k_{q}, \sum_{o'=0}^{\theta-q'} \pi_{o'} + \sum_{o'=\theta-q'+1}^{\theta} k_{q-q'+(\theta-o')} \, \pi_{o'} \,. \end{aligned} \tag{S28}$$

The transition probabilities Π of the occupancy are more complex and require the estimation of how many customers leave the vehicle at the stop. Since we cannot track individual customers, we introduce two new random variables, only tracking one step explicitly and treating all customers who drive longer than one stop identically:

- L_1 denotes the number of customers that were picked up at the last stop and are dropped off at the current stop.
- L_∞ denotes the number of customers in the vehicle for more than one stop that are dropped off at the current stop.

We again write the transition probability as the marginal probability

$$\begin{aligned} \Pi_{o,o'} &= P(O = o \,|\, O' = o') \\ &= \sum_{q'=0}^{\infty} \sum_{l_1=0}^{\min\left[q', \theta - o'\right]} \sum_{l_\infty=0}^{o'} P(O = o \,|\, L_1 = l_1, L_\infty = l_\infty, Q' = q', O' = o') \, P(L_1 = l_1 \,|\, L_\infty = l_\infty, Q' = q', O' = o') \\ &\qquad \times P(L_\infty = l_\infty \,|\, Q' = q', O' = o') \, P(Q' = q' \,|\, O' = o') \,. \end{aligned} \tag{S29}$$

Similar to the above calculation, we take $P(Q' = q' \,|\, O' = o') = p_{q'}$.

Since there is no difference between customers, both L_1 and L_∞ follow Binomial distributions $B(l_1; \min\left[q', \theta - o'\right], p_1)$ and $B(l_\infty; o', p_\infty)$, respectively, where the second argument described the total number of customers that could be dropped off (i.e. that were picked up at the last node or that remained in the vehicle) and the third argument denotes topology-dependent drop-off probabilities measured from simulations. Alternatively, estimates for these probabilities could be obtained by counting neighboring nodes, assuming customers travel along shortest paths in the limit of large fleet size.

Given all other quantities, the occupancy follows deterministicly as

$$O = O' + \min\left[Q', \theta - O'\right] - L_1 - L_\infty \,. \tag{S30}$$

Inserting these probabilities into Eq. (S29) and evaluating the δ operators results in the final expression

$$\Pi_{o,o'} = \sum_{q'=0}^{\infty} \sum_{l_1=l_1^{\min}}^{l_1^{\max}} B\left(l_c; \min(\theta - o',\, q'), p_1\right) B\left(o' - o + \min(\theta - o',\, q') - l_c; o', p_\infty\right) p_{q'} \tag{S31}$$

with

$$\begin{aligned} l_1^{\min} &= \max(0,\, \min(\theta - o',\, q') - o) \\ l_1^{\max} &= \min(\theta - o',\, q') - \max(0,\, o' - o) \,. \end{aligned} \tag{S32}$$

We numerically compute the distributions $\mathbf{p}$ and π by iterating Eq. (S23) with the transitions proabbilities Eq. (S28) and (S31) for 100 times starting from a random initial distribution. We re-normalize the distributions in each step such that they describes a mean occupancy x, accurate in the limit of large fleet sizes and high efficiencies. To facilitate the numerical implementation, we cut off the queue length distribution at $q_{\max} = 50$. The distribution of the occupancy is naturally bounded by the capacity θ.

A. Estimation of p_{delay}

Following the same approach as above, we compute p_{delay} via

$$p_{\text{delay}}^{\infty} = \frac{E(D)}{E(Z)} \tag{S33}$$

with

$$E(Z) = x' \tag{S34}$$

from Eq. (S21) and

$$E(D) = \sum_{d=0}^{\infty} d\, P(d) = \sum_{d,z,q,q'=0}^{\infty} \sum_{o,o'=0}^{\theta} d\, P(d\,|\,q,z,o,o',q')\, P(q\,|\,z,o,o',q')\, P(z\,|\,o,o',q')\, P(o\,|\,o',q')\, P(o'\,|\,q')\, P(q')\,. \tag{S35}$$

Substituting all conditional probabilities

$$\begin{aligned}
P(d\,|\,q,z,o,o',q') &= \begin{cases} \delta_{d,\,z-(\theta-o)} & \text{if} \quad q' \le \theta - o' \\ \delta_{d,\,z-(\theta-o-(q'-(\theta-o')))} & \text{if} \quad \theta - o' \le q' \le 2\theta - o' - o \\ \delta_{d,\,z} & \text{if} \quad q' \ge 2\theta - o' - o \end{cases} \\
P(q\,|\,z,o,o',q') &= \begin{cases} \delta_{q,\,z} & \text{if} \quad q' \le \theta - o' \\ \delta_{q,\,z+(q'-(\theta-o'))} & \text{if} \quad q' > \theta - o' \end{cases} \\
P(z\,|\,o,o',q') &= k_z \\
P(o\,|\,o',q') &= \pi_o \\
P(o'\,|\,q') &= \pi_{o'} \\
P(q') &= p_{q'}
\end{aligned} \tag{S36}$$

and evaluating the sum analogously to the calculation in the minimal model, we arrive at the estimate

$$\begin{aligned}
p_{\text{delay}}^{\text{est}} = \frac{1}{x'} \sum_{o,o'=0}^{\theta} \pi_o\, \pi_{o'} \Bigg[& \sum_{z=\theta-o+1}^{\infty} (z - (\theta - o))\, k_z \sum_{q'=0}^{\theta-o'} p_{q'} \\
& + \sum_{q'=\theta-o'+1}^{2\theta-o-o'-1} \sum_{z=2\theta-q'-o-o'+1}^{\infty} (z + q' - 2\theta + o + o')\, k_z\, p_{q'} \\
& + x_{\text{eff}} \left(1 - \sum_{q'=0}^{2\theta-o'-o-1} p_{q'} \right) \Bigg] ,
\end{aligned} \tag{S37}$$

which we evaluate as the minimal model results numerically.

This calculation with a deterministic inter-arrival time distribution (Poisson distributed new arrivals Z) underlies the results presented in the inset of Fig. 4b in the main manuscript. A comparison between estimations with equidistant arrivals and exponentially distributed inter-arrival times is shown in Fig. S1.

B. Estimating $\mathbf{E}_\infty(\mathbf{B}_{\text{eff}})$.

In order to compare a constrained system with its unconstrained equivalent, we calculate the effective fleet size $B_{\text{eff}} = (1 - p_{\text{delay}})\, B$. To compute $E(\mathcal{G}, B_{\text{eff}}, x, \theta = \infty)$ at potentially non-integer values B_{eff}, we interpolate between efficiencies as follows.

For each $(\mathcal{G}, x)$, we select several fleet sizes B' and find the corresponding efficiencies $E(\mathcal{G}, B', x, \theta = \infty)$ by simulation. In search of a regression function $E^{\sim}_{\infty}(\mathcal{G}, \cdot, x, \theta = \infty)$ that fits this data, a multilayer perceptron with hidden layers of sizes $(5, 10, 5)$ and a tanh activation function has been trained with an $L2$ regularization using `scikit-learn`. The values on the vertical axis of Fig. **??**b are the outcomes of inserting B or B_{eff} into this function $E^{\sim}_{\infty}$. While this approach is not strictly necessary to interpolate the efficiency function, it has the additional advantage of compensating statistical fluctuations from the measured efficiencies.

C. Estimating B_{req}.

To estimate the required fleet sizes, we fix the graph $\mathcal{G}$, the vehicle capacity θ, the vehicle velocity v and the request rate λ. We compute an estimate of the universal scaling function for various loads x via regression of all model topologies using a neural network of four hidden layers of sizes $(80, 20, 10, 5)$ and a tanh activation function. We start with an estimate B_{req} and the resulting estimate for the delay probability p_{delay} to compute the efficiency via the regression of the universal scaling function. Since the load x changes as we vary the fleet size, we interpolate linearly between values of the scaling parameter $B_{1/2}(\mathcal{T}, x)$ if required. We vary the fleet size B_{req} until the predicted efficiency is equal to the target efficiency E_{tar}.

[1] N. T. J. Bailey, On queueing processes with bulk service, J. R. Stat. Soc. B **16**, 80 (1954).

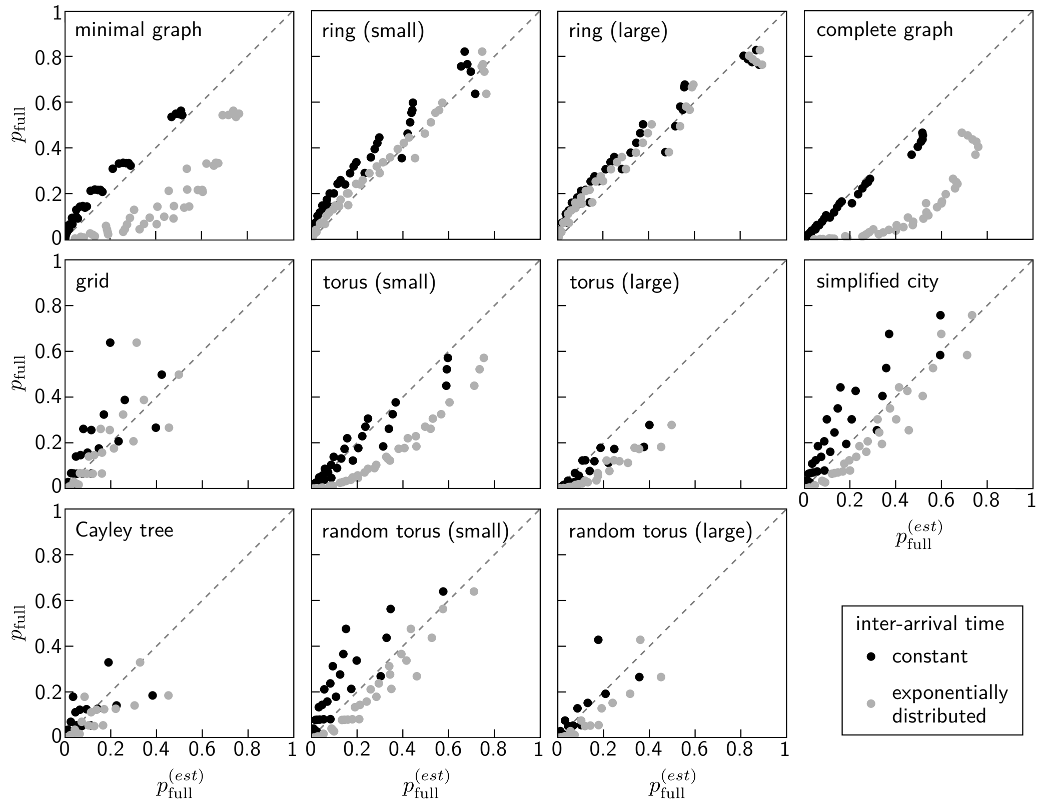

Supplementary Figure S1. **Estimation of the delay probability** p_{delay}**.** Black dots represent estimates of p_{delay} assuming equidistant arrivals of vehicles, gray dots represent the same estimate assuming an exponential inter-arrival time distribution. See Methods in the main manuscript for details on the settings and simulations.
